# Functions for Retinoic Acid-Related Orphan Receptor Alpha (RORα) in the Activation of Macrophages During Lipopolysaccharide-Induced Septic Shock

**DOI:** 10.3389/fimmu.2021.647329

**Published:** 2021-03-09

**Authors:** Emily Hams, Joseph Roberts, Rachel Bermingham, Padraic G. Fallon

**Affiliations:** School of Medicine, Trinity College Dublin, Trinity Biomedical Sciences Institute, Dublin, Ireland

**Keywords:** RORA, macrophage, inflammation, LPS, mice

## Abstract

The transcription factor Related Orphan Receptor Alpha (RORα) plays an important role in regulating circadian rhythm, inflammation, metabolism and cellular development. Herein we show that in the absence of functional RORα in mice there is reduced susceptibility to LPS-induced endotoxic shock, with selective decreases in release of pro-inflammatory cytokines. Treatment of mice with a RORα selective synthetic inhibitor also reduced the severity of LPS-induced endotoxemia. The reduction in responses in Rora deficient mice was associated with an alterations in metabolic and pro-inflammatory functions of macrophages, both *in vivo* peritoneal macrophages and *in vitro* generated bone marrow derived macrophages. Using LysM^Cre^*Rora*^fl/sg^ mice the reduced susceptibility to LPS was shown to be specific to *Rora* expression in the macrophages. This study identifies that *Rora*-mediated regulation of macrophages impacts on the pro-inflammatory responses elicited by LPS.

## Introduction

The transcription factor retinoic acid receptor-related orphan receptor alpha (RORα) is a member of the nuclear hormone receptor superfamily, which provides a bridge between hormonal, nutritional, and pathophysiological signaling and gene regulation. In mammals there are three major isotypes, RORα, RORβ, and RORγ, with each of these able to form multiple variants through alternative splicing (1, 2). RORα itself has been identified as having roles in neural development, metabolism, cellular differentiation, immune regulation and circadian rhythm. Indeed, staggerer mice (*Rora*^sg/sg^), which express a truncated form of the RORα protein due to a spontaneous mutation in the *Rora* gene, show aberrant immune responses (3–6).

Indeed, studies on RORα in the context of metabolism have demonstrated the impact of RORα in signaling pathways associated with lipid and glucose metabolism, alongside influencing the low-grade chronic inflammation that is often a hallmark of metabolic disease (4, 7–9). We have previously demonstrated that *Rora*-expressing myeloid-derived macrophages play an integral role in metabolic dysfunction associated with obesity (8). While the expression of RORα in macrophage subsets is now widely observed, there is some disparity in the apparent role of RORα within these cells (10, 11). Many studies implicate RORα in the inflammatory response to the TLR agonist LPS through its ability to down-modulate NF-κB signaling and impair activation of the NLRP3 inflammasome (3, 6, 10). Furthermore, studies have shown *Rora* is required for the release of other pro-inflammatory cytokines, such as IL-6 and TNF, from macrophages upon activation (12), while in Kupffer cells, RORα drives the activation of alternatively activated macrophages (13), which collectively suggest an anti-inflammatory role for RORα. Conversely, other studies demonstrate a more pro-inflammatory role for RORα, with studies in the mouse retina demonstrating an upregulation in *Socs3*, and associated decrease in pro-inflammatory cytokines, in the absence of *Rora* (14). In addition, there is evidence that in the context of adipose tissue inflammation, as associated with obesity, RORα is capable of driving inflammation. Indeed, it has been demonstrated that RORα can drive endoplasmic reticulum stress, which promotes adipose tissue inflammation (11), and in the absence of *Rora*-expressing myeloid cells, there is a decrease in inflammatory macrophages accumulating in the adipose tissue in a model of obesity (8). These disparate roles for RORα in similar inflammatory context suggest that the impact of RORα may be modified by tissue- and cell-specific factors.

In this study the role for RORα in the genesis of LPS-induced endotoxic shock in mice was examined, with a focus on identifying the immune cells involved. Using *Rora*^sg/sg^, *Rora*^sg/sg^ bone-marrow chimera (BMC) mice, synthetically blocking the action of RORα, and LysM^Cre^*Rora*^fl/sg^ mice, modeling both ubiquitous and myeloid-cell specific deletion, we demonstrate that RORα can promote recruitment and activation of a myeloid-derived pro-inflammatory macrophage population, which impacts upon the severity and extent of LPS-induced endotoxic shock.

## Materials and Methods

### Animals

**Figure 1:**
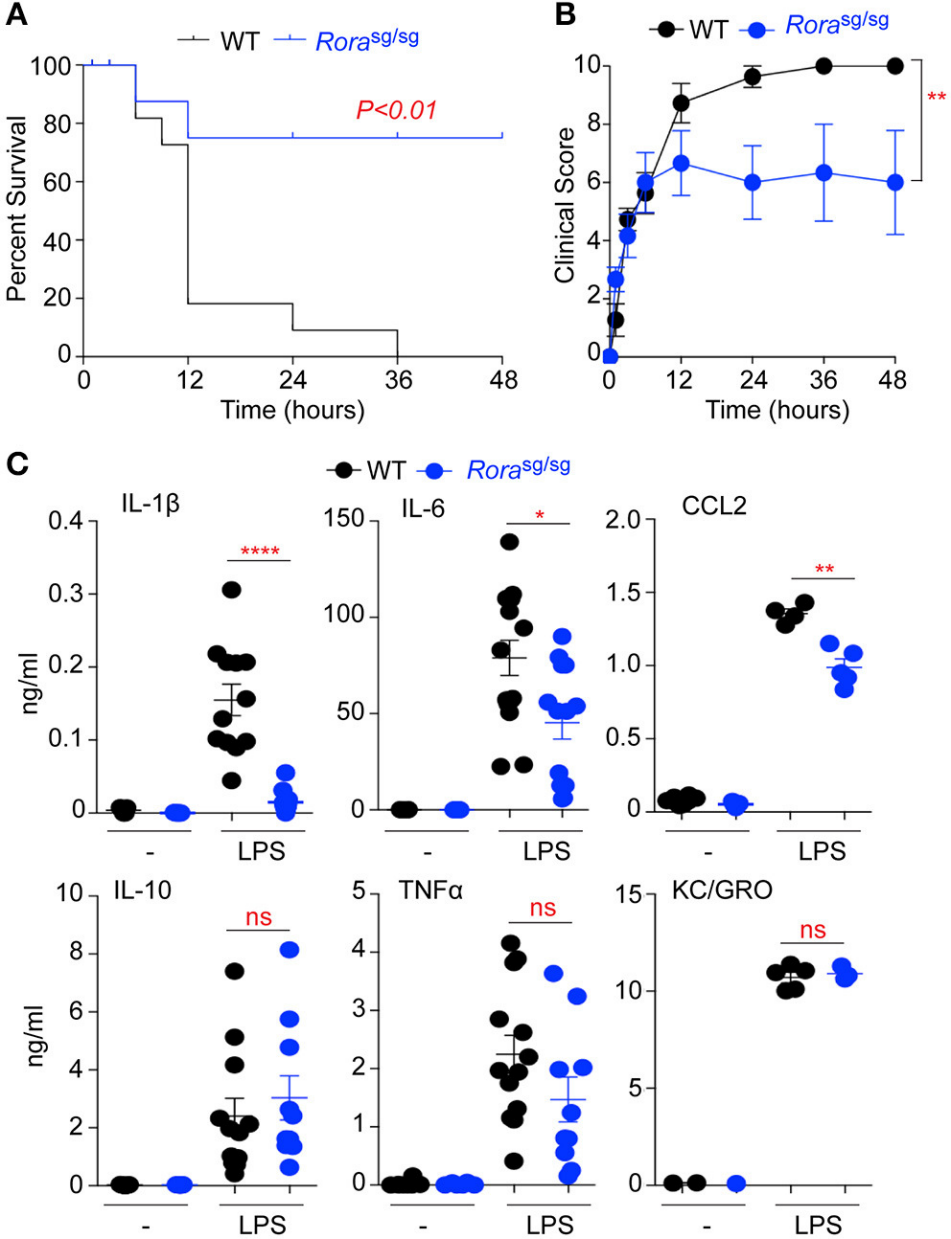
*Rora*^sg/sg^ mice show decreased morbidity in LPS-induced endotoxic shock. WT C57BL/6 (*n* = 12) and *Rora*^sg/sg^ C57BL/6 (*n* = 10) mice were challenged with 10 mg/kg UP-LPS i.p. Mice were monitored periodically for 48 h post LPS injection and death **(A)** and clinical score **(B)** recorded. Blood was collected from naïve animals (-) and those 3 h post LPS injection (LPS; *n* = 5 per strain), serum cytokines and chemokines were determined by ELISA **(C)**. ^*^P < 0.05, ^**^P < 0.01, ^****^P < 0.00001.

**Figure 2:**
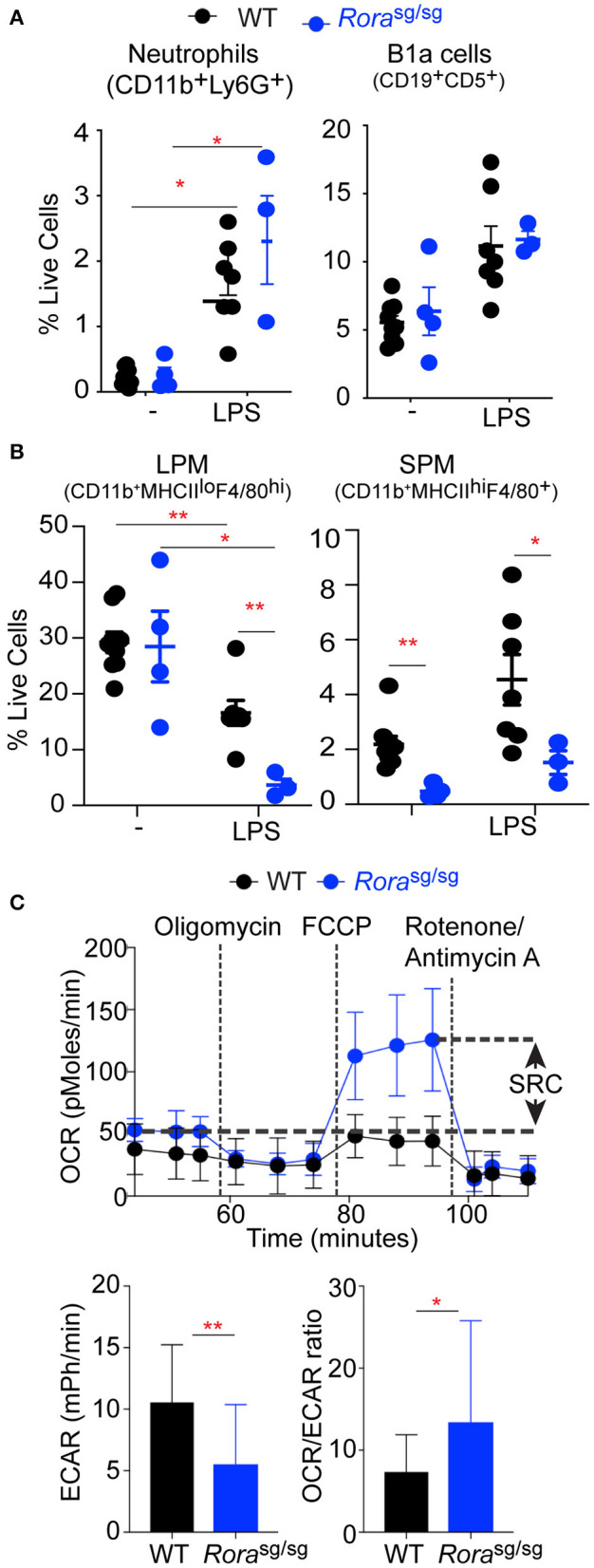
*Rora*^sg/sg^ mice have an altered macrophage response in a naïve state and in response to LPS. Flow cytometric analysis of **(A)** neutrophils (CD11b^+^Ly6G^+^), B-1cells (CD19^+^CD5^+^) and **(B)** large peritoneal macrophages (LPM; CD11b^+^F4/80^hi^MHCII^lo^) and small peritoneal macrophages (SPM; CD11b^+^F4/80^+^MHCII^hi^) isolated from peritoneum of 3–9 naïve WT or *Rora*^sg/sg^ mice and WT and *Rora*^sg/sg^ mice 3 h post injection of 0.2 mg/kg UP-LPS. **(C)** Extracellular flux analysis of naïve peritoneal macrophages from WT (*n* = 3 mice, 8 replicates per mouse) and *Rora*^sg/sg^ (*n* = 3 mice, 6 replicates per mouse) mice showing SRC, basal ECAR and OCR:ECAR ratio. ^*^P < 0.05, ^**^P < 0.01.

**Figure 3:**
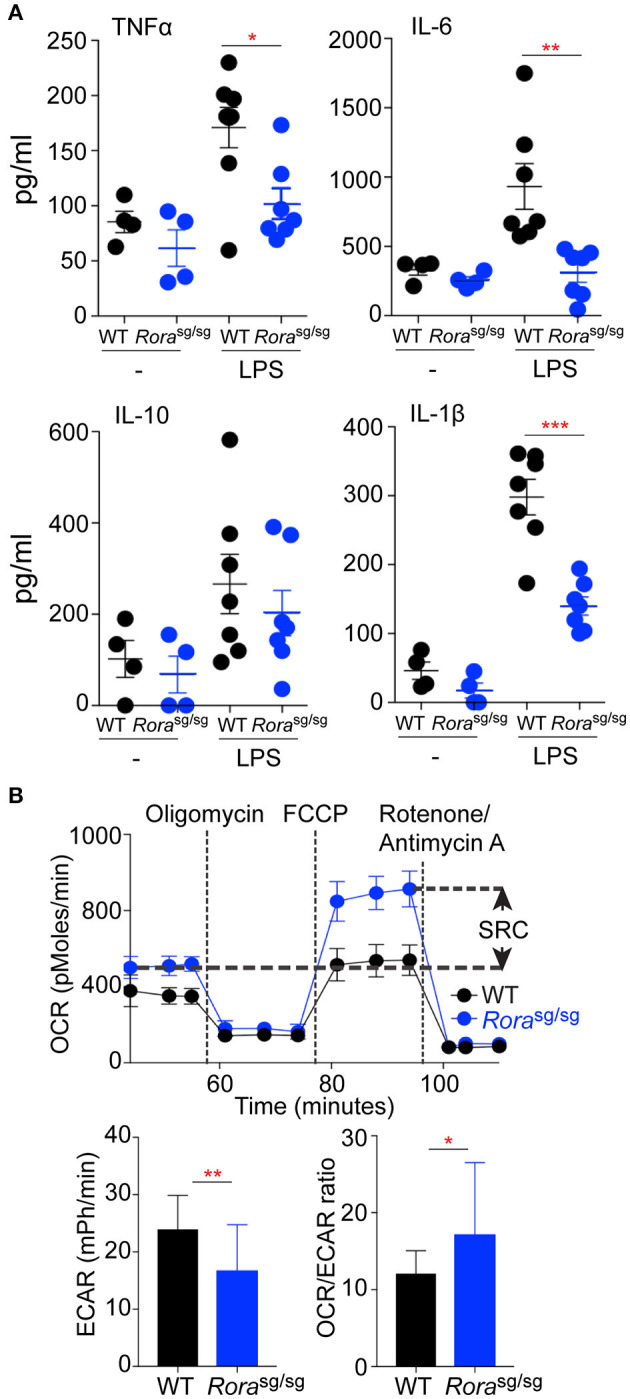
Macrophages derived from *Rora*^sg/sg^ mice display a reduction in the *in vitro* inflammatory response to LPS. Peritoneal macrophages were isolated from naïve WT (*n* = 4, 1 replicate for media control, 2 replicates for LPS stimulated) and *Rora*^sg/sg^ (*n* = 4, 1 replicate for media control, 2 replicates for LPS stimulated) mice and cultured with UP-LPS (200 ng/ml) for 6 h **(A)**. **(B)** Extracellular flux analysis of BMDM from WT (*n* = 3 mice, 8 replicates per mouse) and *Rora*^sg/sg^ (*n* = 3 mice, 6 replicates per mouse) mice showing SRC, basal ECAR and OCR:ECAR ratio. ^*^P < 0.05, ^**^P < 0.01, ^***^P < 0.0001.

**Figure 4:**
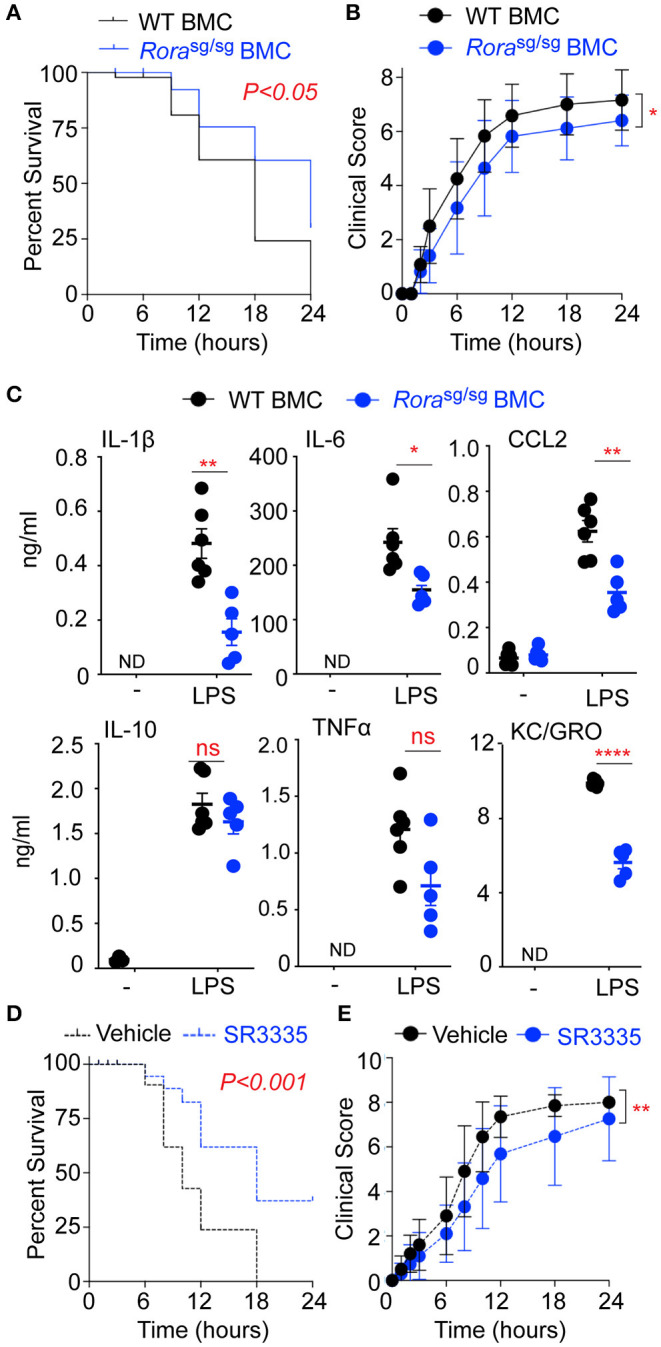
RORα BMC mice have decreased disease severity and inflammatory cytokine release in response to LPS. BMC mice were generated *via* transfer of bone marrow isolated from WT and *Rora*^sg/sg^ mice into irradiated WT recipients (*n* = 12 and *n* = 17, respectively). Mice were challenged with 10 mg/kg UP-LPS i.p. and were monitored periodically for 24 h post LPS injection and death **(A)** and clinical score **(B)** recorded. Blood was collected from naïve BMC animals (-) and treated mice 3 h post LPS injection (LPS; *n* = 6 per group), serum cytokines and chemokines were determined by ELISA **(C)**. WT mice were treated with vehicle control (*n* = 20) or SR3335 (*n* = 19) at a dose of 15 mg/kg for days i.p. On day 8 mice were challenged with 10 mg/kg ultra-pure LPS i.p. and were monitored periodically for 48 h post LPS injection and death **(D)** and clinical score **(E)** recorded. ^*^P < 0.05, ^**^P < 0.01, ^****^P < 0.00001.

**Figure 5:**
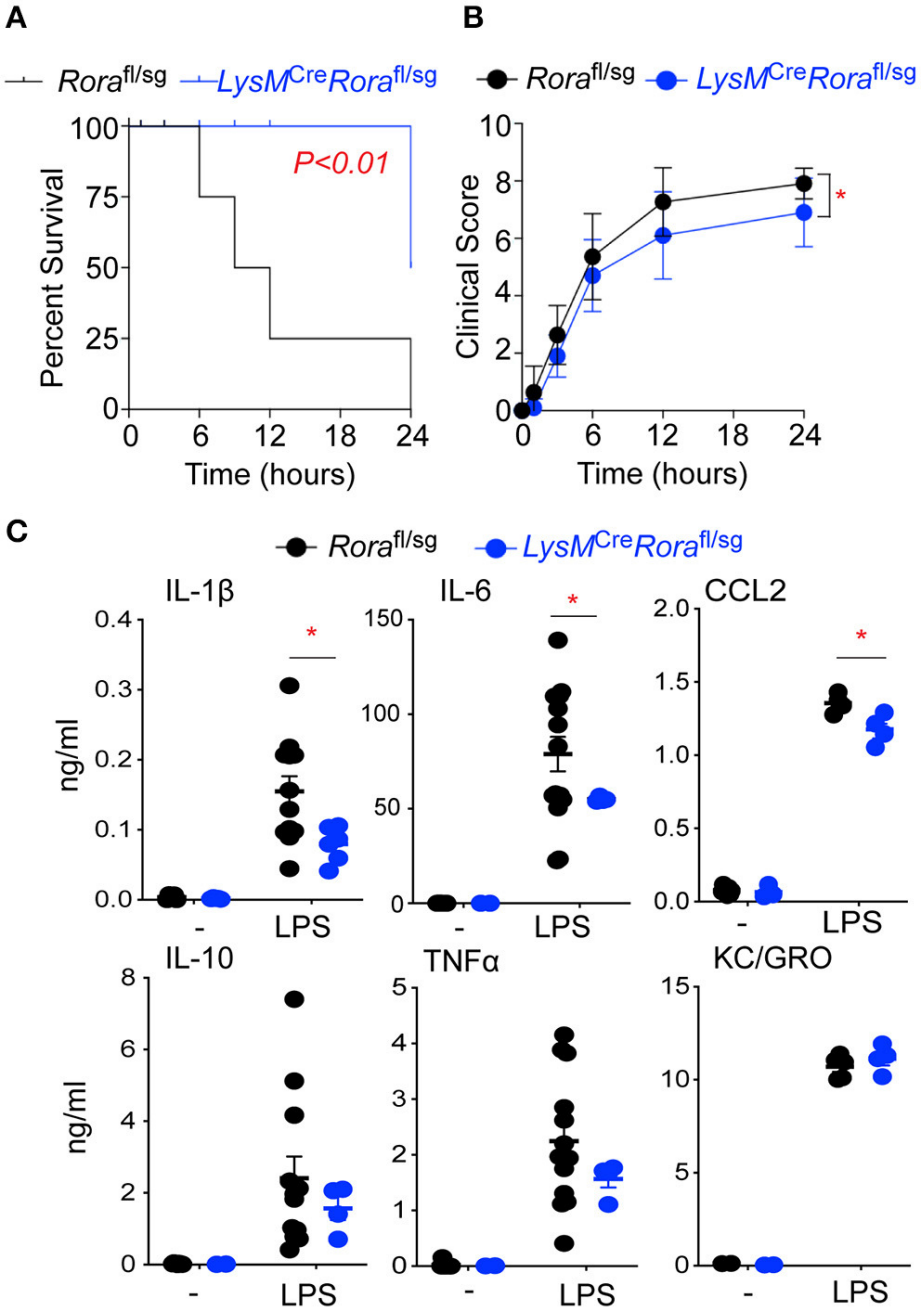
LysM^Cre^*Rora*^fl/sg^ mice show decreased disease severity and an impaired ability to generate an inflammatory response in a model of endotoxic shock. *Rora*^fl/sg^ (*n* = 11) and LysM^Cre^*Rora*^fl/sg^ (*n* = 10) mice were challenged with 10 mg/kg ultra-pure LPS i.p. Mice were monitored periodically for 24 h post LPS injection and death **(A)** and clinical score **(B)** recorded. Blood was collected from naïve *Rora*^fl/sg^ (*n* = 9) and LysM^Cre^*Rora*^fl/sg^ (*n* = 5) mice (-) and *Rora*^fl/sg^ (*n* = 12) and LysM^Cre^*Rora*^fl/sg^ (*n* = 6) 3 h post LPS injection (LPS) and serum cytokines and chemokines determined by ELISA **(C)**. ^*^P < 0.05.

C57BL/6J, staggerer *Rora* spontaneous mutant (JAX strain number 002651; *Rora*^*s*^^g/sg^) and *B6.SJL-Ptprc*^*a*^*Pepc*^*b*^*/BoyJ* (JAX strain number 002014; CD45.1^+^) mice were purchased from Jackson Laboratories (Bar Harbor, MD, USA). Adult 6–10 weeks old female mice were used in all experiments. Conditional *Rora* floxed (*Rora*^fl/fl^; Lexicon Pharmaceuticals, USA) and *Rora*^*s*^^g/sg^ mice were crossed with *Lyz2*^tm1(cre)Ifo^ (JAX strain number: 004781; *LysM*^Cre^) mice to generate *LysM*^Cre^*Rora*^fl/sg^ animals with a conditional deletion of *Rora* in cells of a myeloid lineage. LysM^Cre^*Rora*^fl/sg^ were used due to the variable levels of Lyz2 in different macrophage populations that may result in incomplete excision as seen in *LysM*^Cre^*IL-4R*α^*flfl*^ mice (15). Animals were housed in a specific pathogen-free facility in individually ventilated and filtered cages under positive pressure. All animal experiments were performed in compliance with the Irish Medicines Board (Figures 1, 2) or Health Product's Regulatory Authority (Figures 3–5) and approved by the Trinity College Dublin's BioResources ethical review board.

It is relevant that due to the importance of RORα in regulating circadian rhythm (16), and the corresponding circadian rhythm known to govern circulating monocytes and thus potentially impacting upon the inflammatory response (17), all experiments on mice and tissue isolations were performed at 10 a.m. to ensure no variations occurred due to alterations in the circadian rhythm due to time differences in experiments.

### LPS-Induced Endotoxic Shock

Endotoxic shock was induced in age-matched male C57BL/6J, *Rora*^sg/sg^ and LysM^Cre^*Rora*^fl/sg^ mice by intraperitoneal injection of ultra-pure lipopolysaccharide (UP-LPS) isolated from *Escherichia coli* (Invivogen, France) at a dose of 10 mg/kg, as previously described (18). At 3 h post-injection, blood was collected *via* submandibular bleed for serum cytokine quantification. Temperature was monitored throughout using subcutaneously implanted temperature transponder chips (Bio Medic Data Systems; IPTT 300). Mice were visually scored, using a scale from 1 to 10, at regular intervals, with criteria dependent upon a combination of body temperature, appearance, condition, and behavioral characteristics. The scoring system was: Score 1, <1°C reduction in body temperature, no other change in condition; Score 2, <1°C reduction in body temperature accompanied by reduced interest in surroundings; Score 3, 1–2°C reduction in body temperature accompanied by a reduced interest in surroundings; Score 4, 1–2°C reduction in body temperature accompanied by piloerection; Score 5, 1–2°C reduction in body temperature accompanied by piloerection and reduced interest in surroundings; Score 6, 1–2°C reduction in body temperature accompanied by piloerection and reduced mobility; Score 7, >2°C reduction in body temperature accompanied by piloerection and reduced mobility; Score 8, >2°C reduction in body temperature accompanied by piloerection and nasal and ocular discharge and reduced mobility; Score 9, >2°C reduction in body temperature accompanied by piloerection and nasal and ocular discharge and vocalization; Score, 10 animals that are moribund. All mice were culled at humane end-points or after 48 h (Figures 1, 2) or 24 h (Figures 4, 5).

Clinical scores were performed blinded with individual mice tracked by the transponder chip code. *Rora*^sg/sg^ mice are visually ataxic and stunted and therefore blinding was not practical. All further analysis of cells and serum was performed blinded.

In addition, a lower dose (0.2 mg/kg) of UP-LPS was used to induce cell trafficking into the peritoneal cavity. Groups of age-matched C57BL/6J and *Rora*^sg/sg^ mice were injected intraperitoneally with 0.2 mg/kg UP-LPS and peritoneal cells collected by lavage after 3 h. Cell expression was quantified by flow cytometry.

### Murine Macrophage Isolation and Culture

Peritoneal macrophages were cultured from naive C57BL/6J and *Rora*^sg/sg^ mice as previously described (18). Briefly, the peritoneal cavity was lavaged with 5 ml ice-cold PBS and the resulting cells plated at 2 × 10^6^ cells/ml in RPMI 1640 supplemented with 2 mM L-glutamine, 100 U/ml penicillin and 100 μg/ml streptomycin and incubated for 2 h at 37°C with 5% CO_2_, and any non-adherent cells removed. Adherent cells were stimulated with media-alone or UP-LPS (200 ng/ml) for 4 h, after which culture supernatants were collected for cytokine analysis.

Bone marrow-derived macrophages (BMDM) were cultured from the tibia and fibula of naive C57BL/6J and *Rora*^sg/sg^ mice as previously described (18). Briefly, cells were flushed from the tibia and fibula to prepare a single cell suspension. After red blood cell lysis, the resultant cells were cultured at 3 × 10^6^ cells/ml in RPMI 1640 supplemented with 2 mM L-glutamine, 100 U/ml penicillin, 100 μg/ml streptomycin and 10% fetal calf serum, macrophage colony-stimulating factor (M-CSF) from L929 mouse fibroblast supernatants at 37°C with 5% CO_2_ for 7 days. Cells were stimulated with media alone or UP-LPS (200 ng/ml) for 4 h, after which culture supernatants were collected for cytokine analysis.

### Bone Marrow Chimera Generation

CD45.1^+^ C57BL/6 recipient mice were irradiated using an X-Ray irradiator (XStrahl CIX3), receiving 9 Gy in two doses (5 Gy and 4 Gy) 3 h apart. Mice were reconstituted with 1 × 10^7^ BM cells from CD45.2^+^ C57BL/6 mice or *Rora*^sg/sg^ mice. To ensure efficient irradiation and reconstitution, expression of CD45.1 vs. CD45.2 was assessed by flow cytometry of the spleen after 8 weeks (Supplementary Figure 1).

### Chemical Inhibition of RORα

Mice were treated with SR3335 (N-[4-[2,2,2-trifluoro-1-hydroxy-1-(trifluoromethyl)ethyl]phenyl]-2-thiophenesulfonamide; Cayman) (Cambridge Biosciences, UK) a synthetic inverse agonist of RORα (19). C57BL/6 mice were treated with vehicle control, or SR3335 at a dose of 15 mg/kg per day i.p. for 7 days (19). On day 8 all animals were injected with UP-LPS i.p. as described above.

### Metabolism Assays

Peritoneal macrophages and BMDM, prepared as above, were analyzed with an XF-24 Extracellular Flux Analyzer (Seahorse Biosciences, Agilent Technologies) to determine oxygen consumption rate (OCR) and extracellular acidification rate (ECAR). Cells cultured at a density of 5 × 10^5^ cells/ml were incubated with non-buffered RPMI supplemented with 25 mM glucose with no CO_2_ for 1 h prior to analysis. Three consecutive measurements were taken at basal conditions and then after the sequential addition of 1 μM oligomycin, 1.5 μM FCCP (fluoro-carbonyl cyanide phenylhydrazone) and 1.25 μM rotenone plus 2.5 μM antimycin A. The OCR and ECAR were measured basally and after the addition of each inhibitor. The spare respiratory capacity (SRC) is determined as the difference between the basal OCR and maximal OCR, after the addition of FCCP (20).

### Cytokine and Chemokine ELISA

Cytokine and chemokine levels were quantified in tissue culture supernatants and serum by sandwich ELISA. IL-6 was quantified using matched antibody pairs from BD Biosciences (Oxford, UK), IL-1β, TNFα, IL-10, GROα, and CCL2 were quantified using the DuoSet ELISA development system from R&D Systems (Abingdon, UK) following the manufacturer's protocol.

### Flow Cytometry

Surface marker expression was assessed by flow cytometry with data collection on a CyAn ADP (Beckman Coulter, High Wycombe, UK) and data analyzed using FlowJo software (Tree Star, OR USA). Cells were isolated from the peritoneal cavity by lavage with ice-cold PBS. Cells were stained with BD Biosciences (Oxford, UK) mAbs; F4/80-FITC (BM8), CD45.2-PE-CF594 (104), SiglecF-AlexaFluor647 (E50-2440), CD3-PECF594 (145-2C11); eBiosciences (Dublin, Ireland) mAbs; CD11b-PerCP (M1/70), F4/80-eFluor450 (BM8), CD5-APC (53-7.3), MHC class II-eFluor 450 (MS/114.15.2); and BioLegend (London, UK) mAbs; Ly6G-APCCy7 (1A8), CD19-PerCP (6D5). Prior to surface staining, cells were incubated with LIVE/DEAD Fixable Aqua stain (Molecular probes, Invitrogen, Dublin, Ireland) to isolate dead cells. Using appropriate controls, quadrants were drawn and data were plotted on logarithmic scale density-plots.

### Statistics

Statistical analysis was performed using GraphPad InStat®. Results are presented as mean +/- SEM. Differences, indicated as two-tailed *P*-value, were considered significant when *P*>0.05 as assessed by unpaired Student's *t*-test with Welch correction applied as necessary. Survival statistics were calculated using log-rank correlation. Area Under Curve (AUC) for the clinical scores of individual mice were determined and difference between group clinical score AUC were analyzed by Student's *t*-test.

## Results

### RORα Promotes Inflammation in a Model of LPS-Induced Endotoxic Shock

RORα has been widely implicated in the control of inflammatory signaling, with many studies demonstrating an anti-inflammatory role for RORα through suppression of IκB, a negative regulator of the NFκB pathway (3, 12). RORα is also integral in the generation of a functional type 2 immune response, through expression in ILC2 (21). To further explore roles for RORα in inflammation *Rora*^sg/sg^ mutant mice, which produce a truncated form of the RORα protein, were challenged with LPS to induce endotoxic shock. Mutant mice had significantly improved survival (*P* < 0.01) after LPS challenge compared to WT C57BL/6 mice (Figure 1A), which was associated with a significant (*P* < 0.01) reduction in clinical signs of endotoxemia (Figure 1B).

Assessment of serum cytokines and chemokines in response to LPS treatment shows significantly (*P* < 0.05–0.0001) decreased levels of the pro-inflammatory IL-1β and IL-6 and the monocyte chemoattractant CCL2 in *Rora*^sg/sg^ mice relative to WT animals (Figure 1C). Interestingly, there was no significant reduction in the serum expression of IL-10, TNFα or the neutrophil chemoattractant KC/GRO in *Rora*^sg/sg^ mice compared to WT animals (Figure 1C). These data show RORα deficient mice have reduced susceptibility to LPS-induced endotoxic shock, with selective alteration in production of pro-inflammatory cytokines and chemokines.

### The Macrophage Populations Are Altered in the Peritoneal Cavity of Rora^sg/sg^ Mice

To further assess the *in vivo* cellular response to LPS, mice were administered a lower dose (0.2 mg/kg) to elicit local activation and cellular recruitment to the peritoneal cavity. In WT animals, the cellular profile in response to LPS can be characterized by an infiltration of neutrophils (CD11b^+^Ly6G^+^; Supplementary Figure 2B), B1a cells (CD19^+^CD5^+^; Supplementary Figure 2D) and small peritoneal macrophages (SPM; CD11b^+^F4/80^+^MHCII^hi^; Supplementary Figure 2C) which temporarily replace the resident large peritoneal macrophage population (LPM; CD11b^+^F4/80^hi^MHCII^lo^; Supplementary Figure 2C). There were no differences in neutrophils and B1a cells in the peritoneum of naive WT and *Rora*^sg/sg^ mice, with comparable influx of both cells following LPS treatment (Figure 2A). However, while there was no differences in the number of resident LPM in naïve WT and *Rora*^sg/sg^ mice, there were significantly (*P* < 0.01) fewer SPM in the peritoneal cavity of naïve *Rora*^sg/sg^ mice (Figure 2B), in accordance with previously published studies (8). In response to LPS the influx of SPM in the peritoneal cavity was comparable between WT and Rora^sg/sg^ animals (Figure 2B). Whereas, there was a significant decrease in the number of LPM in the peritoneal cavity of *Rora*^sg/sg^ mice compared to WT animals after intraperitoneal LPS treatment (Figure 2B).

Alterations in recruited monocytes has also been observed in the adipose tissue, with myeloid-derived macrophages pro-inflammatory macrophages significantly decreased in the absence of RORα (8). In both the obesity model and in response to LPS, there is reduced response to the inflammatory stimuli in the absence of RORα. The response to inflammatory stimuli can be influenced by the metabolic state of the macrophage populations themselves, with inflammatory macrophages relying on aerobic glycolysis while anti-inflammatory macrophages, like utilizing fatty acid oxidation to fulfill their energy requirements, reflecting M1-like and M2-like cells, respectively (20, 22). To further examine the differences in the macrophage populations in *Rora*^sg/sg^ mice, the metabolic status of peritoneal macrophages isolated from naive WT and *Rora*^sg/sg^ mice were assessed by extracellular flux analysis. We compared the oxygen consumption by unstimulated peritoneal macrophages from WT and *Rora*^sg/sg^ mice and show enhanced OCR and increased SRC in macrophages isolated from *Rora*^sg/sg^ mice compared to WT macrophages (Figure 2C), which is indicative of increased oxidative phosphorylation, as associated with M2-like macrophages (20). Conversely, macrophages isolated from WT mice showed significantly (*P* < 0.01) increased ECAR, which is indicative of increased reliance upon anaerobic glycolysis and a switch toward an M1-like phenotype (Figure 2C). Additionally, comparison between the ratio of OCR to ECAR shows a significantly (*P* < 0.05) altered commitment toward oxidative phosphorylation and anaerobic glycolysis in cells from *Rora*^sg/sg^ mice (Figure 2C). The characterization of the metabolic profile of peritoneal macrophages from *Rora*^sg/sg^ mice suggests a reduction in an M1 phenotype in the absence of RORα.

These data indicate that in the absence of functional RORα mice develop alterations in the activation or recruitment of macrophages within the peritoneum. Resident LPM and monocytes recruited to the peritoneal cavity and SPM have different origins, with resident macrophages derived from an embryonic precursor and maintained by self-proliferation and renewal, while circulating monocytes are myeloid-derived (23–25). The observation that solely the myeloid-derived macrophage population are altered in naive *Rora*^sg/sg^ mice suggests that during the embryonic stage where the resident cells are initially seeded, there is no apparent role for RORα, however, RORα does appear to impact on the recruitment and polarization of infiltrating monocytes into the peritoneal cavity. Indeed, circulating levels of CCL2, a chemokine well-defined as a recruitment signal for monocytes (26), are significantly decreased in *Rora*^sg/sg^ mice (Figure 1C).

### Macrophages Derived From Rora^sg/sg^ Mice Display a Reduction in the Inflammatory Response to LPS *in vitro*

To further address the diminished inflammatory response in *Rora*^sg/sg^ mice, *in vitro* assessment of macrophage populations was undertaken. There was a selective reduction in cytokine release from peritoneal macrophages isolated from naive *Rora*^sg/sg^ mice (Figure 3A), which mirrored the *in vivo* cytokine responses. As the altered macrophage profile was shown in myeloid-derived macrophage populations in *ex vivo* conditions, the metabolic profile of BMDM from WT and *Rora*^sg/sg^ mice was assessed. While macrophages from both strains display an OCR profile associated with oxidative phosphorylation as indicative of an alternatively activated phenotype—which would be expected as these macrophages were expanded *in vitro* with M-CSF—the BMDM from *Rora*^sg/sg^ mice showed an increased SRC and decreased ECAR relative to cells from WT mice which is in agreement with a bias toward an anti-inflammatory phenotype in the absence of RORα (Figure 3B). This metabolic analysis suggesting an inherent bias toward an anti-inflammatory phenotype in the absence of *Rora*, is interesting supporting previous studies have focused on the anti-inflammatory role of RORα (3, 6, 10, 12, 27). While the signaling pathways elicited by RORα are anti-inflammatory, these data suggest that in the absence of functional RORα there is an alteration in the myeloid compartment that results in a reduction in pro-inflammatory macrophages, which results in the observed diminished pro-inflammatory response.

### Deletion of Functional RORα in the Myeloid Compartment Reduces Inflammatory Cytokine Release and Mortality in a Model of LPS-Induced Endotoxic Shock

In mutant mice, the ubiquitous deletion of functional RORα results in an amelioration of inflammation and a reduction and delay in LPS-associated morbidity (Figure 1). However, due to the wide-ranging roles of RORα in cerebellar development and lipid and glucose metabolism, the mutant *Rora*^sg/sg^ mice are stunted in growth, ataxic, and show increased mortality (28). To circumvent the impact of ubiquitous deletion of RORα, BMC mice were generated using bone marrow isolated from *Rora*^sg/sg^ mice, with a control BMC reconstituted with bone marrow from WT mice. It should be noted that *Rora*^sg/sg^ BMC and WT BMC were phenotypically comparable. *Rora*^sg/sg^ BMC had significantly (*P* < 0.05) decreased mortality (Figure 4A) and improved clinical score (Figure 4B) in response to LPS-induced endotoxic shock relative to WT BMC. Furthermore, the reduced sensitivity to LPS was associated with a concomitant decrease in pro-inflammatory cytokines and chemokines in serum of *Rora*^sg/sg^ BMC mice (Figure 4C).

To further explore roles for RORα endotoxic shock, mice were treated with SR3335, a synthetic RORα selective inverse agonist (19), for 7 days prior to the induction of LPS-induced endotoxic shock. Treatment of mice with SR3335 significantly improves mortality (Figure 4D) and clinical score (Figure 4E) from LPS-induced shock confirming that in this model blocking RORα reduces the severity of disease.

### Deletion of RORα in Myeloid Cells Results in Decreased Inflammation in a Model of LPS-Induced Endotoxic Shock

To validate the role of *Rora* expressing macrophages in the inflammatory response, we generated LysM^Cre^*Rora*^fl/sg^ mice, where the *Rora* gene is excised in cells expressing the *Lyz2* gene, which includes macrophages. When *Rora*^fl/sg^ or LysM^Cre^*Rora*^fl/sg^ mice were injected with LPS, LysM^Cre^*Rora*^fl/sg^ mice had significantly (*P* < 0.01) increased survival (Figure 5A), associated with improved clinical score (Figure 5B) when compared to *Rora*^fl/sg^ animals. In addition, mice with myeloid deletion of *Rora* had significantly (*P* < 0.05) reduced elevations in serum levels of IL-1β, IL-6, and CCL2, but not IL-10, TNF-a or KC/GRO after LPS treatment (Figure 5C). These results are all comparable to the disease progression and severity observed following LPS treatment of *Rora*^sg/sg^ and *Rora*^sg/sg^ BMC mice, suggesting that it is macrophage expression of *Rora* that is responsible for the impact of RORα on the progression of inflammation in this acute model.

## Discussion

RORα is a transcription factor functional in many aspects of neural function, cellular development, immune regulation, metabolism and circadian rhythm. In this study we demonstrate a novel role for RORα in pro-inflammatory response, through myeloid cell expression with the capacity to regulate initiation, recruitment and metabolism of macrophage populations, which impacts upon LPS-induced shock. Studies presented herein provide evidence supporting a role for RORα in promoting a pro-inflammatory macrophage phenotype, with a diminished response to LPS *in vivo* and *in vitro* in the absence of RORα. The pro-inflammatory macrophage specific functions for RORα is interesting considering previous studies have focused on the anti-inflammatory role of RORα through its ability to upregulate IκB and inhibit signaling through the NF-κB pathway (3, 12, 27). Indeed, a previous study has shown increased LPS-induced inflammation in *Rora*^sg/sg^ mice in a model of acute lung injury (6). Furthermore, using the cecal ligation and puncture model of septic shock, an increase in RORα expression at both the mRNA and cytosolic protein level was observed in the heart tissue of septic mice, which was associated with the inhibition of NFκB induced by melatonin, suggesting that a functional RORα response is necessary for the initiation of an innate response against inflammation (10). In accordance with previous studies, we also note decreased expression of IκB in macrophages isolated from *Rora*^sg/sg^ mice (data not shown). Indeed, while the signaling pathways impacted upon by RORα induces an anti-inflammatory role, the ability of RORα to alter the macrophage repertoire leads to the observed pro-inflammatory phenotype we have observed, as exemplified in reduced LPS responses in LysM^Cre^*Rora*^fl/sg^ animals. Furthermore, while other studies have demonstrated an anti-inflammatory role for RORα in macrophages, it is important to note that this study focuses on primary macrophages isolated from animals deficient in functional RORα, while previous studies have focused on genetic manipulation of immortalized cells (6, 29, 30).

*Rora*^sg/sg^ mice are associated with a stunted and ataxic phenotype, which may confound the response to inflammatory stimuli. BMC reconstituted with bone marrow of *Rora*^sg/sg^ mice, have been used previously to generate ILC2-deficient animals (31). *Rora*^sg/sg^ BMC mice were utilized to circumvent the associated phenotypic concerns with relying upon results derived from *Rora*^sg/sg^ mice in isolation. Whilst *Rora*^sg/sg^ BMC mice did indeed show significantly reduced mortality compared to WT BMC animals when exposed to LPS, the level of significance of this effect was reduced in these animals compared to the *Rora*^sg/sg^ mice. Although we see limited impact of *Rora* expression in resident macrophages (8), we cannot rule out a potential role for these cells, or indeed other *Rora*-expressing stromal cells. Indeed, using a synthetic RORα inverse agonist, which targets RORα ubiquitously, we were able to recapitulate the reduction in mortality and decreased clinical response to LPS-induced endotoxic shock that was observed in both *Rora*^sg/sg^ mice, with an increased level of significance to that observed in *Rora*^sg/sg^ BMC mice. Interestingly we see a significant decrease in KC/GRO in LPS challenged *Rora*^sg/sg^ BMC mice when compared to challenged WT BMC, which was not apparent in LPS challenged *Rora*^sg/sg^ mice. This possibly occurs as a result of *Rora*-expressing hematopoietic cells impacting on neutrophil recruitment, in a manner that is not observed, or is compensated against, in *Rora*^sg/sg^ animals. This reduction in KC/GRO may impact on the overall response to LPS, with the associated decrease in neutrophil recruitment in part responsible for the decreased protection against LPS-induced shock in these animals.

We have demonstrated that peritoneal macrophages express only low levels of *Rora*, with higher *Rora* expression in infiltrating monocyte-derived macrophages (8). Herein we demonstrate a reduction in pro-inflammatory cytokine release from macrophages deficient in functional RORα. Furthermore, we demonstrate a marked difference in the metabolism of macrophages isolated from *Rora* deficient mice, with both peritoneal macrophages from naive mice and BMDM from *Rora*^sg/sg^ mice showing an increased reliance upon oxidative phosphorylation to meet their energy requirements. Further studies are warranted to explore in more detail the altered metabolic phenotype in *Rora* deficient macrophages. These data spanning characterization of macrophage by function and metabolic profile collectively suggest that *Rora* regulates the activation of macrophages to influence the pro-inflammatory response.

RORα has been identified as a transcription factor critical for the development of a number of immune cell populations, including Th17 cells, regulatory T cells, ILC2, ILC3, and macrophage populations (8, 21, 32–34). While RORα may promote intercellular anti-inflammatory signaling pathways, its ability to shape the immune cell repertoire and promote recruitment and activation of pro-inflammatory macrophages demonstrates that RORα also promotes inflammation. These data demonstrate new functions for RORα in macrophage activation and function that is relevant to the associations of *RORA* with inflammatory disease in man.

## Data Availability Statement

The original contributions presented in the study are included in the article/Supplementary Materials, further inquiries can be directed to the corresponding author.

## Ethics Statement

The animal study was reviewed and approved by Trinity College Dublin's BioResources Ethical Review Board.

## Author Contributions

EH and PF: conceptualization and funding acquisition. EH, JR, and RB: methodology and investigation. EH: writing—original draft. All authors: writing—review and editing.

## Conflict of Interest

The authors declare that the research was conducted in the absence of any commercial or financial relationships that could be construed as a potential conflict of interest.

## Supplementary Material

Supplementary Figure 1 Flow cytometry of blood of bone marrow chimeric (BMC) mice. Representative flow cytometry plots showing CD45.1^+^ or CD45.2^+^ cells in the spleen of a CD45.2^+^ *Rora*^sg/sg^ donor mouse, a CD45.1^+^ recipient mouse and a *Rora*^sg/sg^ BMC mouse.

Supplementary Figure 2 Generation of Gating strategy for cells isolated from the peritoneal cavity after LPS treatment. Peritoneal exudate cells were collected by lavage in sterile PBS 3 h after LPS treatment. **(A)** Initial gating strategy to remove dead cells, cell doublets and eosinophils (F4/80^lo^SiglecF^hi^). **(B)** Identification of neutrophils (F4/80^−^Ly6G^+^FSC^hi^). **(C)** Identification of small peritoneal macrophages (SPM; CD11b^+^F4/80^+^MHCII^hi^), and large peritoneal macrophages (LPM; CD11b^+^F4/80^hi^MHCII^+^). **(D)** Identification of B1a cells (FSC^lo^SSC^lo^CD3^−^CD19^+^CD5^+^).
