# Supplementary figures and images for: Functions for Retinoic Acid-Related Orphan Receptor Alpha (RORα) in the Activation of Macrophages During Lipopolysaccharide-Induced Septic Shock
Source: Front Immunol. 2021 Mar 9;12:647329. doi: 10.3389/fimmu.2021.647329 (PMC7986717; doi:10.3389/fimmu.2021.647329)

Figure S1.

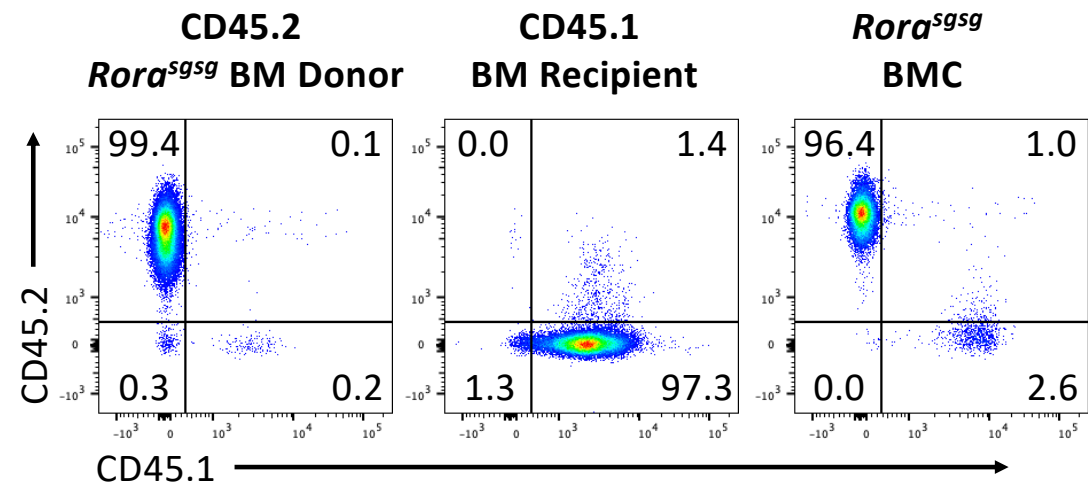

Supplement: Supplementary Figure 1 — Flow cytometry of blood of bone marrow chimeric (BMC) mice. Representative flow cytometry plots showing CD45.1+ or CD45.2+ cells in the spleen of a CD45.2+ Rorasg/sg donor mouse, a CD45.1+ recipient mouse and a Rorasg/sg BMC mouse. [file Image_1.pdf]

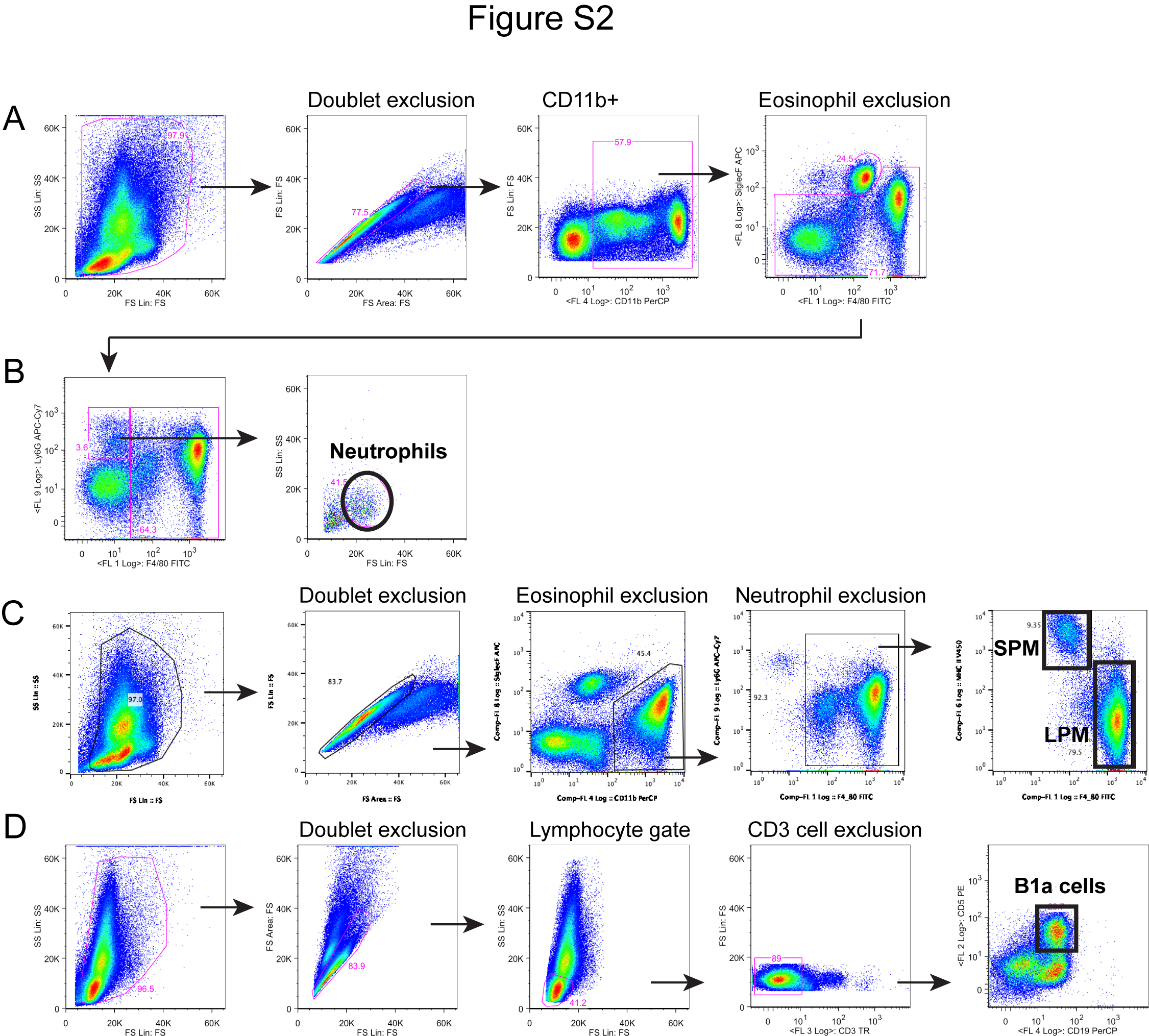

Supplement: Supplementary Figure 2 — Generation of Gating strategy for cells isolated from the peritoneal cavity after LPS treatment. Peritoneal exudate cells were collected by lavage in sterile PBS 3 h after LPS treatment. (A) Initial gating strategy to remove dead cells, cell doublets and eosinophils (F4/80loSiglecFhi). (B) Identification of neutrophils (F4/80−Ly6G+FSChi). (C) Identification of small peritoneal macrophages (SPM; CD11b+F4/80+MHCIIhi), and large peritoneal macrophages (LPM; CD11b+F4/80hiMHCII+). (D) Identification of B1a cells (FSCloSSCloCD3−CD19+CD5+). [file Image_2.TIF]
